# Inhibition and paradoxical choice

**DOI:** 10.3758/s13420-023-00584-2

**Published:** 2023-05-05

**Authors:** Valeria V. González, Aaron P. Blaisdell

**Affiliations:** Department of Psychology, University of California, 1285 Franz Hall, Los Angeles, CA 90095-1563 USA

**Keywords:** Inhibition, Counterconditioning, Extinction, Choice

## Abstract

The present study evaluated the role of inhibition in paradoxical choice in pigeons. In a paradoxical choice procedure, pigeons receive a choice between two alternatives. Choosing the “suboptimal” alternative is followed 20% of the time by one cue (the S+) that is always reinforced, and 80% of the time by another cue (S-) that is never reinforced. Thus, this alternative leads to an overall reinforcement rate of 20%. Choosing the “optimal” alternative, however, is followed by one of two cues (S3 or S4), each reinforced 50% of the time. Thus, this alternative leads to an overall reinforcement rate of 50%. González and Blaisdell (2021) reported that development of paradoxical choice was positively correlated to the development of inhibition to the S- (signal that no food will be delivered on that trial) post-choice stimulus. The current experiment tested the hypothesis that inhibition to a post-choice stimulus is causally related to suboptimal preference. Following acquisition of suboptimal preference, pigeons received two manipulations: in one condition one of the cues in the optimal alternative (S4) was extinguished and, in another condition, the S- cue was partially reinforced. When tested on the choice task afterward, both manipulations resulted in a decrement in suboptimal preference. This result is paradoxical given that both manipulations made the suboptimal alternative the richer option. We discuss the implications of our results, arguing that inhibition of a post-choice cue increases attraction to or value of that choice.

## Introduction

When choosing between two different amounts of a reward, all else being identical, organisms prefer the larger amount (Neuringer, 1967). Under some circumstances, however, hungry organisms will choose a smaller over a larger amount of food, which can be considered paradoxical. One example of this is presenting animals with the choice between two options that differ in the overall probability of food reward as well as in the level of uncertainty with respect to the outcome: Choice of the lower-probability of reward (e.g., 20%) alternative is immediately followed by one of two post-choice stimuli, one that signals that food will be delivered (S+) and the other that signals that no food will be delivered on that trial (S-), both signals lasting 10 s. This alternative is often referred to as the “suboptimal” option. Choice of the higher-probability of reward (e.g., 50%) alternative is immediately followed by one of two post-choice stimuli, S3 or S4, each lasting 10 s and signaling food on half of the trials. This alternative is often referred to as the “optimal” option. Despite the difference in reinforcement rate between the alternatives, pigeons, starlings, and sometimes rats prefer the suboptimal but informative option (Cunningham & Shahan, 2019; Stagner & Zentall, 2010; Vasconcelos et al., 2015; for a review, see González et al., 2023). Most accounts of this suboptimal choice propose that paradoxical choice is due to the subject ignoring the S-, thereby overweighting the S+ (McDevitt et al., 2016; Zentall, 2016). Empirical support for this account comes from evidence that increasing the proportion of S- trials to 90% fails to impact suboptimal preference (Fortes et al., 2016), as well as the S- failing to pass a negative summation test of conditioned inhibition after preference for the Info alternative has emerged (Laude et al., 2014).

González and Blaisdell (2021) pointed out methodological issues with the study by Laude et al. (2014) that weakens their claim, and further provided empirical evidence that preference for the suboptimal alternative develops in lock-step with Pavlovian inhibitory properties of the S- as measured in a summation test, in which responding to an excitatory cue presented simultaneously with a putative conditioned inhibitor is compared to responding to the excitatory cue presented alone. In this study, the responses to the excitor were lower when presented together with S-, showing evidence of Pavlovian inhibition. Moreover, the strength of the paradoxical choice effect was positively correlated with the strength of S- inhibition across individuals. Although these data are correlational, they suggest a causal relation between them. Perhaps the development of preference for the suboptimal alternative depends on the development of inhibition to the S- terminal stimulus. In the suboptimal choice task, animals prefer the option followed by excitatory and inhibitory cues, S+ and S-, that predict the presence and absence of rewards, respectively; cues that reduce uncertainty about the outcome. Previous studies show that information is rewarding (e.g., Bar-Anan et al., 2009; Golman & Loewenstein, 2018). Thus, preference for the suboptimal alternative might result from its being followed by informative cues that reduce uncertainty. If this is the case, then both post-choice cues (i.e., the S+ and the S-) should play a role in driving preference for the suboptimal alternative. There is a general agreement that the S+ plays a role in suboptimal preference (González et al., 2023; Zentall, 2016), thus, our focus is on the disputed role of the S- on suboptimal preference.

The current study was designed to test whether inhibition plays a causal role in suboptimal preference. First, if inhibitory properties of the non-reinforced S- cue induce a preference for the suboptimal alternative, then removing its inhibitory properties should reduce preference for that option, and thereby increase preference for the optimal alternative. Second, if suboptimal preference is driven in part by the inhibitory properties of the S-, then imbuing one of the optimal alternative cues, S4, with inhibitory properties should increase preference for the optimal alternative.

To assess the causal role of inhibition on suboptimal preference, pigeons were first trained on a paradoxical choice procedure until they developed strong and stable suboptimal preference. Then, we conducted two manipulations. One involved the partial reinforcement of S- (50% instead of 0%), which should imbue it with some excitatory value and counteract its inhibitory properties (counterconditioning). Although the manipulation increases the overall reinforcement rate associated with the suboptimal alternative – in fact, it changes it into the richer option, we predict that preference for the suboptimal alternative should decrease.

The other manipulation involved the extinction of S4 (from 50% reinforcement to 0%), which should produce inhibition of extinction (Bouton, 2004; Denniston & Miller, 2003). With extinction of S4, the optimal alternative becomes suboptimal in terms of overall reinforcement rate. Nevertheless, we predict that imbuing S4 with inhibitory properties will increase preference for the optimal alternative. This would also be a paradoxical effect.

Furthermore, extinction of S4 provides an interesting opportunity to observe the nuanced role of inhibition on suboptimal choice. If we allow a sufficiently long delay, say 2 weeks, following extinction of S4, we should observe spontaneous recovery from extinction (Pavlov, 1927). If extinction of S4 caused a reduced preference of the suboptimal alternative due to S4 acquiring inhibitory properties, then the waning of inhibition over a long delay (i.e., spontaneous recovery of S4’s excitation) should result in recovery of suboptimal preference. We test this hypothesis by presenting pigeons with choice trials again after a 2-week delay following the extinction of S4.

By contrast, if inhibition is not necessary to develop a preference for the suboptimal alternative, as suggested by Laude et al. (2014), then partially reinforcing the S- (therefore removing the inhibitory properties) or extinguishing S4 (therefore embedding it with inhibition) should either have *no effect* on preference for the suboptimal alternative or perhaps strengthen preference for the suboptimal alternative.

## Method

### Subjects

Eight adult homing pigeons (*Columba livia*) from Double-T Farms were used. Six of the pigeons had experience with an intelligence test battery (i.e., a set of tasks similar to human tasks used to measure IQ, including discrimination and reversal, working memory, and simple reaction time, but were naïve with respect to the current procedure). Two pigeons had experience with another version of the suboptimal task with different stimuli than those used in the current study 3 years prior to the present study. For all animals, stimuli were selected to minimize transfer from prior experience. Subjects were individually housed in steel home cages with metal wire mesh floors in a vivarium. They were maintained at 80% of their free-feeding weight, with free access to water and grit while in their home cages. Testing occurred during the light portion of the 12-h light-dark cycle.

### Apparatus

The experiment was conducted in a flat-black Plexiglas chamber (38 cm wide × 36 cm deep × 38 cm high). Stimulus events were controlled by computer connected to an LCD color monitor (NEC MultiSync LCD1550M). The bottom edge of the viewing window was 13 cm above the chamber floor. Pecks to the LCD monitor were detected by an infrared touchscreen (Carroll Touch, Elotouch Systems, Fremont, CA, USA) located on the front panel. A custom-built food hopper (Pololu, Robotics and Electronics, Las Vegas, NV, USA) was in the center of the front panel, its access hole flush with the floor. The hopper delivered 5-s to 6-s access to mixed grain as reward. A personal computer operating Windows 10 OS controlled the experimental events and recorded all data.

### Stimuli

**Fig. 1:**
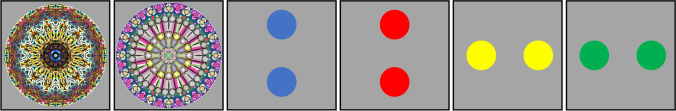
Example of stimuli presented during the task. *Note*. The kaleidoscopic stimuli served as Initial-Link (IL) stimuli. The double-dot stimuli served as Terminal-Link (TL) stimuli. All stimuli were counterbalanced across subjects

Six stimuli were used, two initial-link and four terminal-link stimuli (see Fig. 1). The initial-link stimuli were large circles with a mandala pattern. The terminal-link stimuli were pairs of circles with the same color (red, green, yellow, or blue), horizontally or vertically aligned. Each mandala or pair of circles stimulus occupied a 100 × 100 pixel square. Stimuli were presented against a gray background, 12 cm apart, on the left and right sides of the screen. Assignment of functional role of each stimulus was pseudorandomly determined across subjects. The procedure was coded using Python.

### Procedure

#### Acquisition

**Fig. 2:**
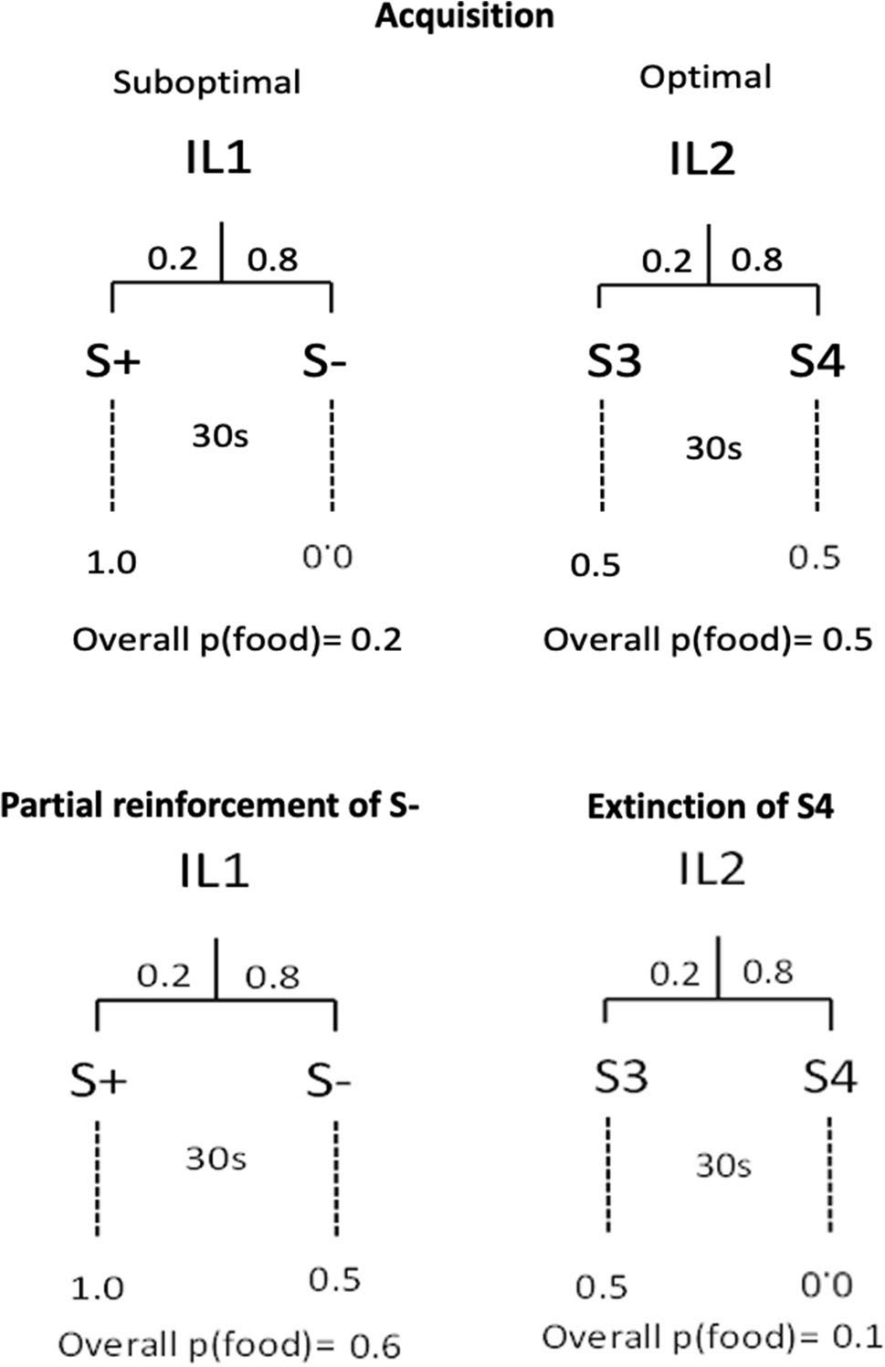
Experimental task. *Note.* The two alternatives and their contingencies are presented. The initial-link stimuli were counterbalanced across animals. The top panel shows the contingencies used during Acquisition for both alternatives. The bottom panel shows the probability associated with S4 during the Extinction phase (right side) and the probability associated with S- during the partial reinforcement phase (left side)

There were two types of trials in each acquisition session of 120 trials, free-choice (40 trials) and forced-choice (80 trials; see trial design in Fig. 2). In a free-choice trial, pigeons were presented with a choice between Initial link (IL) stimulus 1 and stimulus 2 (IL1 and IL2) presented on the left and right sides of the screen evenly counterbalanced within each session. A single peck to one of the initial link stimuli removed them from the display and presented the terminal link stimulus for 30 s on the same side of the screen as the selected initial link stimulus had been located. Choice of IL1, the suboptimal alternative, was followed on 20% of the trials by S+, and on 80% of the trials by S-. The S+ was always followed by food upon its termination, while the S- was never followed by food during acquisition. Choice of IL2, the optimal alternative, was followed by S3 on 20% of the trials and by S4 on 80% of the trials, each reinforced on 50% of the trials in each session. A black screen presented for 10 s served as the intertrial interval (ITI). On forced-choice trials, pigeons were presented with only one of the initial link stimuli, IL1 or IL2 (see Fig. 2) with left/right location also counterbalanced within each session. In both types of trials, the stimulus presentation after the choice followed a sampling without replacement method. Sessions lasted until all trials completed or 120 min elapsed.

The acquisition phase consisted of a minimum of 16 successful sessions (i.e., the pigeon completing at least half of the trials in a session) and continued until the following stability criteria were met: (a) there was no strictly increasing or decreasing trend in the proportion of choices in the last three sessions, and (b) the difference between the highest and lowest preference in the last three sessions did not differ by more than 20%. Once stability was reached, half the pigeons (Jubilee, Durrell, Vonnegut, and Luigi) received (a) Extinction of S4, the Choice Test and, 2 weeks later, the Spontaneous Recovery Test, and then (b) Reacquisition Training, Partial Reinforcement of S-, and the Choice Test. The remaining pigeons (Athena, Estelle, Mario, and Wenchang) received (a) Partial reinforcement of S- followed by the Choice Test, and then (b) Reacquisition Training, Extinction of S4, the Choice Test, and, 2 weeks later, the Spontaneous Recovery Test. After the first manipulation of S4 or S-, all pigeons experienced the *Reacquisition* phase before starting the remaining manipulation.

#### Extinction of S4

Pigeons received six sessions of 80 trials of IL2 only. As during acquisition, a single peck on IL2 caused it to be removed from the display and immediately followed on 20% of the trials by the presentation of S3, which ended in reinforcement half of the time, or on 80% of the trials by the presentation of S4, which never ended in reinforcement (i.e., extinction). S3 and S4 were presented for 30 s.

#### Partial reinforcement of S-

Pigeons received six sessions consisting of 80 trials of only IL1. A single peck to IL1 was followed by its removal from the display and presentation of either the S+ or S-. On 80% of these trials, S- was presented for 30s and half of these trials ended in reinforcement (i.e., partial reinforcement of the S-). On the remaining trials IL1 was followed by the 30-s S+, which always ended with reinforcement.

#### Choice test

Following the six sessions of Extinction of S4 or Partial reinforcement of S-, pigeons received two sessions each containing ten free-choice trials, which were the same as acquisition trials, except none of these trials ended in reinforcement.

#### Spontaneous recovery test

Following the two Choice test sessions after Extinction of S4, pigeons remained in their home cages for 2 weeks without receiving any experimental manipulations. Following this 2-week period, pigeons received one session of ten free-choice trials as described for the Choice test.

#### Reacquisition training

Following the first testing round (Choice test or Spontaneous recovery test), pigeons were put back in the original training contingencies for five sessions to re-establish the level of preference for the suboptimal alternative obtained at the end of Acquisition and to prepare for the second manipulation.

### Data analysis

Preference was defined as the number of choices to the suboptimal alternative divided by the total number of free-choice trials completed. The data were analyzed using a repeated-measures ANOVA (RM-ANOVA) in which Sessions was the within-subject factor. When a significant main effect was found, post hoc analysis using Holm corrections were implemented.

## Results

**Fig. 3:**
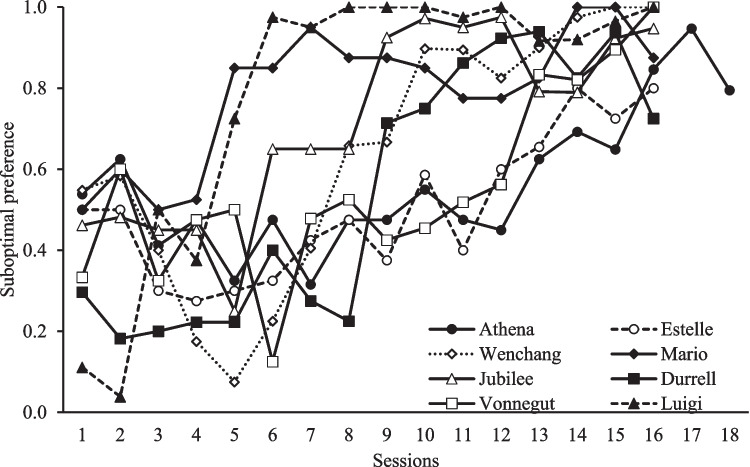
Acquisition data. *Note*. The suboptimal preference (number of suboptimal choices divided by the total of choice trials) is shown by bird across sessions of Acquisition

Figure 3 shows that, by the end of acquisition, all pigeons developed a preference above 70% for the suboptimal alternative. To compare the acquisition of preference across all pigeons, we averaged the first three sessions of Athena to equate the number of sessions across pigeons. A RM ANOVA found a main effect of Session, *F*(15,105) = 11.815, *p* < .001, *η*^2^ = .628 showing a successful acquisition of the suboptimal choice preference.

**Fig. 4:**
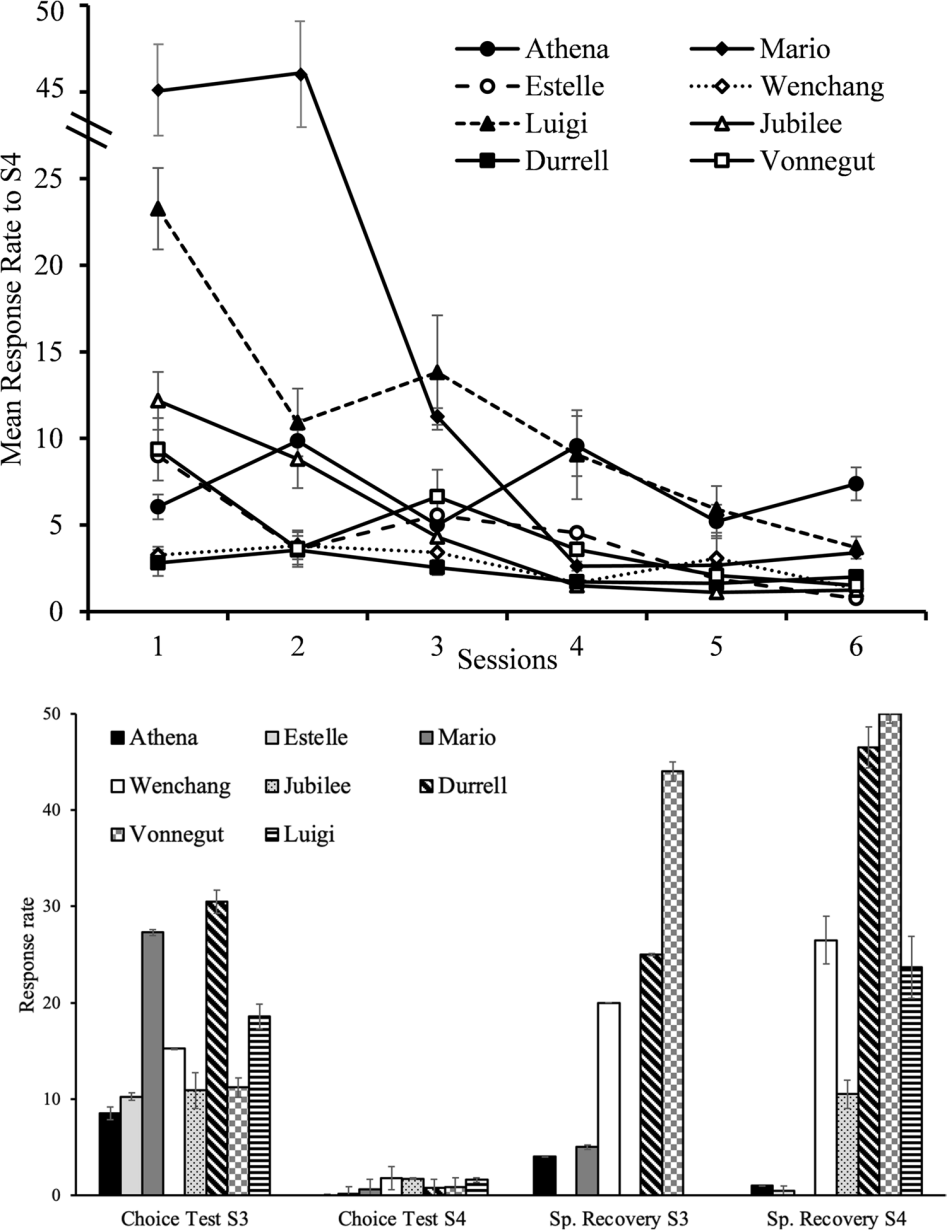
Mean response rate during Extinction of S4 (top) and first Choice Test for S3 and S4 and Spontaneous Recovery Test (bottom). Top: The average number of responses during the 30-s presentation of the cues (i.e., the response rate) during the six sessions of Extinction of S4 are presented by pigeon. Bottom: The average response rate of S3 and S4 in the first Choice test and the Spontaneous recovery test are presented by pigeon. Notice that *Estelle, Jubilee*, and *Luigi* did not have any presentation of S3 and *Mario* did not have a presentation of S4 during the Spontaneous recovery test. Error bars depict standard errors of the mean (SEMs)

Preliminary analysis found no order effect, and thus we analyzed each manipulation separately pooling across all birds. Figure 4 shows the average response rate during the six sessions of Extinction of S4 (top) and to S4 and S3 during the first Choice test and the Spontaneous recovery test (bottom). It is important to note that animals are not required to peck during the delay stimulus; therefore, changes in response rate to the Pavlovian cue might not occur. Nevertheless, there is a general tendency for response rate to S4 to decrease during extinction except for Athena. In contrast, almost all pigeons increased their responses to S4 from Choice test to Spontaneous recovery, whereas response to S3 remained on a similar level.

**Fig. 5:**
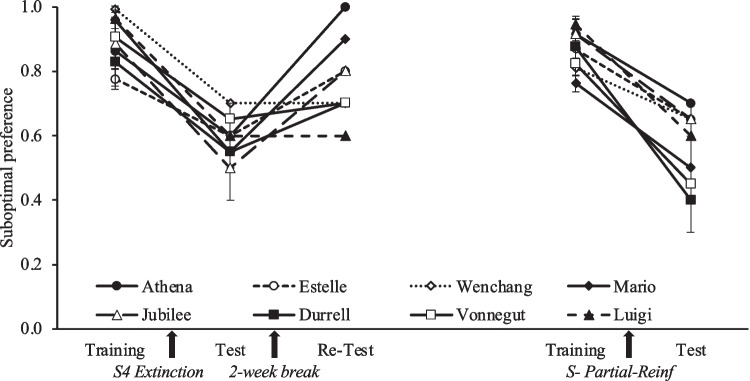
Comparison between Acquisition/reacquisition (training) and test sessions. *Note*. Acquisition corresponds to the average of the last three session. Test is the average of both tests. Error bars depict the standard errors of the mean (SEMs). The black arrows indicate the manipulation that occurred between conditions

Figure 5 shows suboptimal preference across all phases of training and testing. The Acquisition/reacquisition data are collapsed across the last three sessions of each phase. The left side of the graph shows how preference changed with the S4 manipulation (Extinction and Spontaneous recovery), whereas the right side shows how preference changed with the S- manipulation (described in the next paragraph). A RM ANOVA was conducted for the manipulation with S4, with the three related phases (Acquisition, Extinction of S4 and Spontaneous recovery) as within-subject factor. We found a significant effect of Phase, *F*(2,14) = 19.255, *p* < .001, *η*^2^ = .733. Post hoc analyses with Holm's method revealed that preference for the suboptimal alternative decreased following the Extinction of S4 (Test < Training, p < .001), but it increased after the Spontaneous Recovery period (Test < Re-Test, p = .005).

**Fig. 6:**
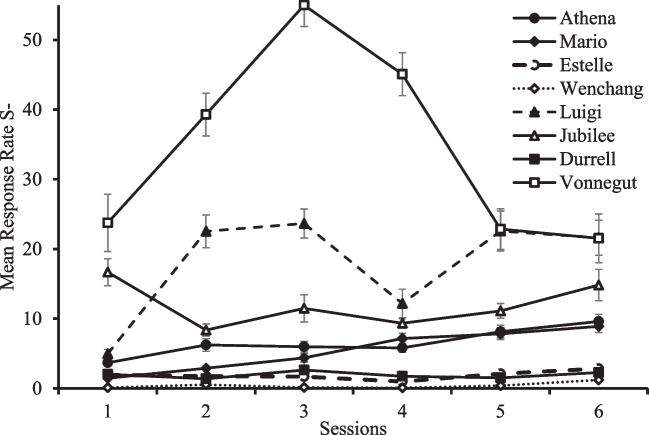
Response rate during Partial reinforcement of S-. *Note*. The average number of responses during the 30-s presentation of the cues (i.e., the response rate) during the six sessions of Partial reinforcement of S- are presented by pigeon. Error bars depict standard errors of the mean (SEMs)

A paired-sample t-test was used to evaluate the effect of partial reinforcement of S-. We found a difference between Acquisition/Reacquisition and the Choice test following partial reinforcement of S- (*t*(7)= 7.973, *p* < .001), in which the preference for the suboptimal alternative decreased after S- become partially reinforced (see right side of Fig. 5). Figure 6 shows the average response rate during the 6 sessions of Partial Reinforcement of S-, in which we observed that only half of the pigeons increased the responses to S- across sessions, perhaps because responses were not required during the duration of the cue.

## Discussion

The present study evaluated the extent to which inhibitory properties of the post-choice cue influence the preference for the suboptimal alternative. We evaluated the role of inhibition with two complementary manipulations: counterconditioning the inhibitory properties of the S- through partial reinforcement, and extinguishing the excitatory properties of S4 through nonreinforcement. Both manipulations resulted in a decrease in the initial preference for the suboptimal alternative, and a corresponding increase in the preference for the optimal alternative. The change in cue-reward contingencies produced by each manipulation increased the relative rate of reinforcement for the suboptimal alternative, effectively making it the richer option. Nevertheless, the preference for that option decreased; a result that can be considered a paradoxical effect of a paradoxical effect. The initial preference for the suboptimal alternative is considered paradoxical because animals show a preference for the leaner option that is followed by informative cues, despite those cues occurring post-choice and therefore having no apparent instrumental utility. Both manipulations here changed the suboptimal alternative to become the richer option by increasing the overall reinforcement rate from 20% to 60% when S- became a partially reinforced cue; or by decreasing the reinforcement rate of the optimal alternative from 50% to 10% when S4 was extinguished. Nevertheless, these manipulations led pigeons to shift their preference away from the initially suboptimal alternative towards the initially optimal alternative. Overall, the results of our study challenge the idea that post-choice cues associated with absence of reinforcement have no impact on decision making involving multiple concurrent choices.

Our study is not exempt from limitations. For example, the manipulations of S+ and S4 were conducted in sessions containing only “forced-choice,” single-option trials. Perhaps conducting sessions without choice trials is what caused the reductions in preference for the suboptimal alternative option and corresponding increase in preference for the optimal alternative. Nevertheless, we think this is unlikely given that a recent experiment by McDevitt et al. (2022) suggested that presenting sessions containing only forced-choice trials (as in our manipulations) facilitates learning the contingencies of the task. Moreover, they found that exposure to sessions containing only forced trials increased suboptimal preference when choice trials were reintroduced, which is the opposite of the change in preference found following our manipulations. Furthermore, the training phase in our experiment followed a ratio of 2:1 of forced versus choice trials. Thus, the extended exposure during the Extinction of S4 and the Partial Reinforcement of S- were not that different from the training phase.

The results presented here might contradict other studies reporting that manipulating the duration or the probability of reinforcement of S- (similar to what was done here) did not change preference (Fortes et al., 2016, 2018). Yet, there are significant differences in how our experiment was carried out compared to those by Fortes et al. We did not manipulate cue durations in our experiment, therefore direct comparisons between those studies and ours can’t be made. Nevertheless, if in their study, animals reached an asymptotic level of learning about the inhibitory properties of S-, then no changes in preference are expected. Thus, ours and their results are consistent.

In contrast, an experiment by Fortes et al. (2018, Experiment 1) manipulated the reinforcement rate of S- across individuals, thereby rendering it a partially reinforced cue rather than an S-. Probability of reinforcement following the “S-” ranged across individuals from 0% (i.e., a true S-), to 37.6% of S- trials. Animals were assigned to different conditions and trained with the different “S-” from the beginning of the experiment. The authors found that the higher the rate of reinforcement of “S-”, the slower the preference for the suboptimal alternative developed. By the end of training, however, all pigeons preferred the suboptimal alternative. In our case, animals first developed a suboptimal preference with the traditional task involving a true S-, and then the contingency of S- was changed through partial reinforcement. Thus, their manipulation was not comparable to ours.

A recent study by Ajuwon et al. (2022) found that the use of explicit cues can facilitate a preference for the suboptimal alternative. In their experiment, three different groups of rats learned the suboptimal choice task: one group had explicit cues for S+ and S- (auditory cues), another had an auditory S+ but a “silent” S-, while the last group had an auditory S- but a “silent” S+. All three groups of rats developed a preference for the suboptimal and informative alternative. However, the group with a silent S+ took longer and showed more variability. This result is not inconsistent with our results. Both show that learning about discriminative cues contributes to choice behavior. That is, Ajuwon et al. show that the identity of the S+ and of the S- each independently contribute to the development of suboptimal preference, while our results focus on the demonstrated role of inhibitory properties of the post-choice cue (whether established during acquisition such as the S-, or through extinction, such as with S4) are one determinant of IL choice.

Our results suggest that information conveyed by the post-choice cue, that is the certainty value of the signal, plays a causal role in suboptimal choice preference. Furthermore, inhibition could be one of the mechanisms by which information affects choice. This does not deny the contributions of S+ in preference, but builds the case that learning about either type of cue (S+ and/or S-) can influence paradoxical preference. The results presented here suggest that information about the presence or absence of outcomes is learned, and excitatory and inhibitory properties are acquired to post-choice cues. We claim that, rather than being ignored, an inhibitory cue can pull preference toward its antecedent initial-link stimulus on a choice trial. In consequence, the results of this experiment suggest that pigeons increase their choice of the optimal alternative when the information conveyed by S4 was increased through inhibition of extinction, or when the information conveyed by S- decreased through partial reinforcement.

We couched our explanation of the results in terms of the information that the cues provided about the outcome. The informational approach is, of course, not the only explanation. Perhaps a simpler explanation, such as contrast, can also account for our results. Various approaches to contrast have been elaborated, especially as applied to paradoxical choice. For instance, the Delta-Sigma model (González et al., 2020a, 2020b) can explain changes in preference as a result of our manipulations in terms of contrast. In this model, the main factor behind suboptimal preference is the contrast between post-choice cues within an alternative – that is, the difference in probability of reinforcement associated with each cue that follows that alternative. In the typical task, the contrast for the suboptimal alternative is 1 (probability of 1 signaled by the S+ minus the probability of 0 signaled by the S-), while the optimal alternative has a contrast of 0 (probabilities of both S3 and S4 are equal to .5). In our study, partially reinforcing S- on a 50% schedule decreased the contrast in the suboptimal alternative from 1 to .5, whereas extinguishing S4 increased the contrast in the optimal alternative from 0 to .5. In both cases, the suboptimal alternative is still the option with higher contrast (.5 vs. 0 and 1 vs. .5, respectively). Nevertheless, as a result of each manipulation, the difference between alternatives decreased from 1 to .5, which can explain the reduction in preference for the suboptimal option.

An alternative approach to contrast was suggested by Case and Zentall (Case & Zentall, 2018), who suggested that the overall reinforcement rate provides the expectation of what will be obtained; but then the post-choice cues create a contrast between what was expected and what was obtained. Therefore, for the suboptimal alternative (with the typical contingencies), the expectation is .2 and the presence of S+ increases the expectation to 1, creating a positive contrast of .8, whereas the presence of S- decreases the expectation to 0, creating a negative contrast of .2 (i.e., -.2). For the optimal alternative there is a contrast of 0, because what is expected and obtained is always .5. For the original approach, the authors claimed that S- did not play any role because the contrast between what was expected (.2) and the presence of S- (0) was only -.2. Therefore, the contrast for S- was minimal and had little to no influence in preference. More recent research suggests, however, that negative contrast might have an impact if it is sufficiently large (Zentall et al., 2019). Applying this argument to the results presented here, we found that, for the partial reinforcement of S-, the expectation of overall reinforcement became .6, therefore the contrast in the presence of S+ is .4 (from .6 to 1), whereas the contrast in the presence of S- is -.1 (from .6 to .5). Although Zentall and collaborators do not specify how different contrasts in a given option are combined, if we assume that (as they do) that a contrast of -.1 is “ignored,” then the overall contrast of the suboptimal alternative is .4. Furthermore, if we assume that the contrasts are added, the overall contrast for the suboptimal alternative is .3 (.4+ -.1). In both cases, the contrast for the suboptimal option is higher than the contrast in the optimal alternative (which remains at 0), but smaller than in the original task (i.e., prior to partial reinforcement of the S-), thus reducing the strength of the preference for the suboptimal option.

Similarly, the contrast for the optimal alternative when S4 is extinguished became .4 (if we assumed the extinguished S4 is ignored) or .3 (if we assumed both contrasts are added). That is, S3 has a contrast of positive .4 (from an expectation of .1 overall reinforcement rate to .5), and S4 has a contrast of negative .1 (from .1 to 0), The suboptimal alternative still has a higher contrast of +.8. In both cases, the contrast still favors a suboptimal preference, but the difference in contrast between options is reduced, which could explain the decrement in preference for the suboptimal option after both manipulations.

Overall, our results seem to challenge the generally accepted idea that non-reinforced cues have no impact on preference in the paradoxical choice paradigm. Further research is needed to resolve the discrepancies between our results and those from prior studies (but see González & Blaisdell, 2021, for an argument on why those earlier studies should be discounted). Nevertheless, we believe the results presented here can motivate researchers to further investigate what is learned about non-reinforced cues, and in particular the role of inhibition on choice. Previous and current results from our lab suggest an associative mechanism for regarding what is learned about post-choice cues (González & Blaisdell, 2021; Trujano et al., 2016) that is based on classic learning theory (Rescorla, 1969). In this associative framework, manipulations that change the inhibitory/excitatory properties have an impact on preference, indicating the important role that the current status of post-choice cues plays at the moment of choice. Evidence showing that the initial choice is connected with the subsequent event has been deeply researched in the “concurrent-chain choice” literature (Grace, 2016). Therefore, we should expect that in the suboptimal choice procedure the task is learned as a whole, with manipulations of post-choice cues affecting subsequent choice.

## Data Availability

The datasets generated during and/or analyzed during the current study are available on figshare.

## Declarations

This research was conducted following the relevant ethics guidelines for research with animals and was approved by UCLA’s institutional IACUC, and in compliance with the APA ethical standards in the treatment of animals.
